# Preparing Emergency Medicine Residents to Disclose Medical Error Using Standardized Patients

**DOI:** 10.5811/westjem.2017.11.35309

**Published:** 2017-12-14

**Authors:** Carmen N. Spalding, Sherri L. Rudinsky

**Affiliations:** * Naval Medical Center San Diego, Bioskills/Simulation Training Center, San Diego, California; † Naval Medical Center San Diego, Department of Emergency Medicine, San Diego, California

## Abstract

**Introduction:**

Emergency Medicine (EM) is a unique clinical learning environment. The American College of Graduate Medical Education Clinical Learning Environment Review Pathways to Excellence calls for “hands-on training” of disclosure of medical error (DME) during residency. Training and practicing key elements of DME using standardized patients (SP) may enhance preparedness among EM residents in performing this crucial skill in a clinical setting.

**Methods:**

This training was developed to improve resident preparedness in DME in the clinical setting. Objectives included the following: the residents will be able to define a medical error; discuss ethical and professional standards of DME; recognize common barriers to DME; describe key elements in effective DME to patients and families; and apply key elements during a SP encounter. The four-hour course included didactic and experiential learning methods, and was created collaboratively by core EM faculty and subject matter experts in conflict resolution and healthcare simulation. Educational media included lecture, video exemplars of DME communication with discussion, small group case-study discussion, and SP encounters. We administered a survey assessing for preparedness in DME pre-and post-training. A critical action checklist was administered to assess individual performance of key elements of DME during the evaluated SP case. A total of 15 postgraduate-year 1 and 2 EM residents completed the training.

**Results:**

After the course, residents reported increased comfort with and preparedness in performing several key elements in DME. They were able to demonstrate these elements in a simulated setting using SP. Residents valued the training, rating the didactic, SP sessions, and overall educational experience very high.

**Conclusion:**

Experiential learning using SP is effective in improving resident knowledge of and preparedness in performing medical error disclosure. This educational module can be adapted to other clinical learning environments through creation of specialty-specific scenarios.

## BACKGROUND

Emergency medicine (EM) is a high-risk clinical learning environment with reported rates of medical errors between 18% 1 to 32%.^2^ Unique challenges such as frequent interruptions, multiple transitions of care, time constraints, simultaneous management of multiple complex patients, decisions based on incomplete information, unfamiliar physician-patient relationship, and a lack of privacy increase the risk of medical errors and create barriers to effective identification and disclosure when errors occur.^3^,^4^

The ability to effectively disclose medical errors (DME) is crucial in EM. The 2010 American College of Emergency Physicians Policy Statement on Disclosure of Medical Errors 5 directs emergency physicians who determine an error has occurred to provide timely information about the error and its consequences to patients and their families. Despite this mandate, a disclosure gap exists in EM. When surveyed, 88% of emergency department (ED) patients in one academic setting desired full disclosure of the error and 63% of patients endorsed teaching physicians error disclosure techniques, honesty, and compassion as educational priorities.^6^ However, a survey of 55 EM residents from two programs demonstrated infrequent, inadequate disclosure to patients and families, occurring in only 28% of cases.^7^

To close the disclosure gap, the Accreditation Council for Graduate Medical Education (ACGME) has called for improved education surrounding DME during residency training. The ACGME Clinical Learning Environment Review (CLER) Pathways to Excellence 8 calls for “hands-on training” of DME, and the 2017 EM program requirements 9 state “residents must receive training in how to disclose adverse events to patients and families [and] should have the opportunity to participate in the disclosure of patient safety events, real or simulated” as a necessary educational component of the Clinical Learning and Working Environment.

Though the use of standardized patients (SP) in DME training has been described in other specialties,^10^–^13^ there is sparse literature addressing DME training using SP specific to the EM clinical environment.^14^ The purpose of the study was to determine if offering this type of training improved EM residents’ ability to DME in the ED setting.

## OBJECTIVES

The primary objective of this course was to provide EM residents education and hands-on training in DME. We estimated that after this training, participants would report improved knowledge, skills, and attitudes surrounding DME in the ED. Specific objectives were as follows:

1. Define what constitutes a “medical error” and “adverse event”
2. Discuss the ethical arguments and professional standards dictating DME
3. Recognize common barriers to effective DME
4. Describe key elements in the effective DME to patients
5. Apply these key elements during a variety of simulated encounters
6. Employ effective communication skills targeted to DME.

This training was developed to address CLER Patient Safety Pathway 7,^8^ which recommends that residents be provided training related to disclosure of safety events within the clinical setting.

## CURRICULAR DESIGN

We designed this course using the “flipped-classroom” model of adult learning. Participants were provided didactic materials 3,4 for review prior to course attendance. These materials were chosen by the clinical subject matter experts (SME) in collaboration with the healthcare resolution SME.

The course was a four-hour session, developed in collaboration with clinical, healthcare resolutions, and healthcare simulation SME. The course consisted of didactic review (30 minutes), video vignette review with debrief (30 minutes) and standardized patient (SP) encounters (2.5 hours). The remaining 30 minutes were dedicated to course evaluation and survey completion. There were five practice SP encounters and one SP encounter for final assessment. During the practice sessions, participants were broken into small groups and rotated through five scenarios. Each vignette took approximately five minutes, after which the small group participated in a debrief lasting approximately 10 minutes, facilitated by the SP. The simulation SME rotated through each station to proctor at least one debriefing session for each case. The course culminated in a standardized scenario, in which each resident interacted with the SP individually, and the SP provided formal written feedback on their performance.

Case scenarios were developed by clinical SME with assistance from healthcare resolutions and healthcare simulation SME. They provided a variety of medical error situations and patient populations. Scenarios included these:

1. Computed tomography ordered on wrong patient with contrast allergy^15^
2. Pneumothorax requiring chest tube placement after central line
3. Wrong patient information (lab result) given to patient and family
4. Failure to review allergies and wrong medication ordered
5. Epinephrine administration IV instead of IM
6. Wrong dose of insulin with hypoglycemic seizure.

The following is an example of the background provided to the residents prior to engaging in the simulation scenario:

### Scenario 5

A 12-year-old boy with history of multiple allergies presents with hives on his face, swollen lips, and is complaining of a hoarse voice and progressive throat tightness after exposure to an unknown allergen at school. He ambulated into the ED and is speaking in full sentences after receiving oral diphenhydramine in triage. Vital signs on presentation are stable. The physician gives the following verbal orders: 0.3 mL of epinephrine 1:1000 IM, methylprednisone 2 mg/kg IV, ranitidine 1 mg/kg IV. The nurse draws up the medications and gives the IV medications first. When she prepares to give the intramuscular epinephrine, she realizes the epinephrine was administered via IV. The patient begins complaining of severe chest pain, appears diaphoretic, and shows abnormal vital signs. The nurse notifies you of the error. A 12-lead electrocardiogram is performed and reveals ST segment elevation. The patient is treated with supplemental oxygen and sublingual nitroglycerin. Reassessment reveals resolution of the angioedema, ST segment elevation, and chest pain. The vital signs stabilize. The patient is admitted to the PICU for observation. The mother is very upset and demands to know what happened.

Assessment of learning included surveys completed by the participant and checklists completed by the SP. The pre- and post-test surveys measured self-efficacy in their confidence and preparedness in performing key skills in DME.^10^ In addition to being trained to their specific roles, SPs were trained by the healthcare simulation SME to provide feedback on each participant’s performance in the final scenario using a published simulation assessment tool.^16^,^17^

## IMPACT/EFFECTIVENESS

Of the 15 post-graduate year 1 and 2 residents who participated in this course, 66% reported prior DME training, of whom only 13% reported the use of simulation. After the course, residents reported increased preparedness in performing several key elements in DME (Table 1) and demonstrated the ability to apply these key elements during a SP encounter (Table 2). Residents reported this training to be valuable, rating the didactic, SP sessions, and overall educational experience very high (mean scores 4.2, 4.5, and 4.4 respectively; Likert scale, 1=not at all useful, 5= very useful). These results suggest experiential learning using SP is effective in improving resident knowledge of and preparedness in performing DME.

## LIMITATIONS

This pilot course introduced a new and important element to EM residency training. While initial results are encouraging, there are important limitations to consider. Due to faculty unavailability, we were unable to allow for multiple raters, which would have allowed for a more objective assessment of the residents’ performance. Residents were offered reading materials prior to the course, and the faculty relied on self-report to determine whether or not the residents reviewed the materials. Many of the metrics were self-reported, which is less robust than more objective data. Although the objective data provided by the SP is more robust, because there was no pre-course scenario, it was difficult to determine whether this training was the sole source of that level of performance, especially considering that many had previous DME training. Despite these limitations, we believe this course provided a meaningful way to allow for safe practice of DME with robust feedback, and faculty and residents both reported the desire for repeated immersive training in this area.

As this was the first iteration of this course, we had only a small number of participants with limited data. However, our results mirror previous studies, reporting improved self-efficacy^10^ and performance^11^–^13^ of DME after SP interactions. Likewise, similar to studies in which educational value was assessed,^10^,^12^,^13^,^18^ our participants reported the training to be beneficial. While a single course offering may not result in long-term retention of these concepts, the most appropriate timeline for refresher training is unclear. We plan to offer this course annually to reinforce these concepts throughout residency training.

Post-simulation debriefing is widely regarded as essential to skill acquisition and retention.^20^ While our course used the trained SP, rather than faculty, to provide feedback during debriefing, previous studies have shown that the SPs can provide accurate assessments of interpersonal communication skills.^21^, ^22^ Additional studies have demonstrated that with proper training, SP scores correlate highly with faculty experts.^23^ The SP training included a 45-minute session on facilitating debriefing using a published debriefing guide^24^ and giving feedback using the TeamSTEPPs feedback model.^25^

## CONCLUSION

Disclosing medical error, regrettably, is a skill that physicians in nearly every medical specialty will be required to perform at some point in their careers. Suboptimal DME can have lasting detrimental effects on patients, their families, and the healthcare team. Experiential learning using SP is a well-documented method for teaching various forms of communication skills^18^, ^19^ and has demonstrated effectiveness in teaching DME in specialties outside of EM.^10^–^14^ Disclosing medical error is a stressful endeavor for EM residents.^7^ Immersive training via SP affords the opportunity to practice a critical and emotionally uncomfortable skill in a safe environment. It is our hope that the general content and format of this course will be replicated in other graduate medical education programs to help future physicians perform this difficult and emotionally charged responsibility.

## Figures and Tables

**Table 1:** Self-efficacy in error disclosure among 15 emergency medicine residents.

“How prepared do you feel to perform each variable during the disclosure of a medical error?”	Score* mean (SD)		Residents improved, n (%)	P value

	Pre	Post		
Know what to include	2.5 (0.6)	4.4 (0.7)	15/15 (100)	p < 0.001
Introduce the topic with a patient	2.9 (1)	4.3 (0.5)	13/15 (87)	p < 0.001
Deal with a patient’s emotional response	3.1 (1)	3.9 (0.6)	10/15 (67)	p = 0.005
Express empathy	3.9 (0.6)	4.2 (0.6)	8/15 (53)	p = 0.05
Respond to a patient’s questions	3.1 (0.7)	3.9 (0.6)	10/15 (67)	p = 0.003
Address patient concerns about consequences of error	2.5 (0.9)	3.9 (0.7)	11/14 (79)	p < 0.001
Deal with legal questions	1.7 (0.7)	2.7 (0.9)	10/15 (67)	p < 0.001
Recognize your own emotions	3.6 (0.9)	4.1 (0.8)	7/14 (50)	p = 0.006
Keep your emotions in check	3.5 (0.8)	4.2 (0.7)	9/15 (60)	p < 0.001

* Score ranges from 1 (lowest; not at all prepared) to 5 (highest; very well prepared), expressed in mean (SD), p-value using paired t-test.

Survey adapted from Bonnema R et al. *J Grad Med Educ.* 2009;1(1):114

**Table 2:** Critical action checklist for key elements in medical error disclosure.

	Score		
		
Critical action	1 = very poor	5 = excellent	Score mean (SD)
Conducts explicit disclosure of error to patient	Does not explicitly explain that an error took place and the patient had suffered as a result	Describes the nature and source of the error and consequences of the error to the patient and/or family members	3.6 (0.6)
Responds forthrightly to patient’s questions about the event	Avoids direct responses to a family member’s question	Responds truthfully to the patient and/or family member’s questions	4.0 (0.4)
Apologizes upfront and early in conversation	Does not apologize up front	Apologizes to the patient and family member at the beginning of the disclosure conversation	4.4 (0.5)
Exhibits general communication skill with the patient	Remains aloof and distant to family member’s emotional distress	Displays verbal and nonverbal empathy and support of the patient and family member	3.9 (0.5)
Conducts blame-free disclosure, acknowledges personal role	Blames a team member in front of the family member	Avoids blaming of other team members, resists patient and/or family members attempts to affix blame	4.1 (0.6)
Offers plans to prevent future errors	Does not address specific plans for preventing future errors	Explains to patient and/or family member what will be done to prevent such errors from occurring in the future	3.8 (0.7)
Plans follow up with patient	Does not offer to follow up with the family member	Offers to follow up with the patient and family member for other potential questions they may have	3.9 (0.6)

Adapted from Kim et al. *Teaching and Learning in Medicine* 2011;23(1):68 and Biberston K et al. Error Communication: Discover barriers, Share best practices and Lead change with simulation. IMSH 2016.
